# The Impact of Purified Granules Sourced from Potato, Maize and Wheat on Disulfide Bond Formation in Urea-Solubilized Glutenin

**DOI:** 10.3390/foods15152732

**Published:** 2026-08-04

**Authors:** Mi Tian, Wenhui Jing, Jiankang Min, Rui Li, Chunrui Wang, Xijun Lian

**Affiliations:** Tianjin Key Laboratory of Food Biotechnology, College of Biotechnology and Food Science, Tianjin University of Commerce, Tianjin 300134, China; tianmi1@tjcu.edu.cn (M.T.); jingwenhui@tjcu.edu.cn (W.J.); minjiankang@tjcu.edu.cn (J.M.); lirui@tjcu.edu.cn (R.L.); wangchunrui@tjcu.edu.cn (C.W.)

**Keywords:** granules, USG, disulfide bonds, FTIR, ^13^C Solid-state NMR

## Abstract

The addition of potato and maize powders to wheat-based dough systems has been identified as a common practice for enhancing the dietary fiber content of cereal products. However, their product quality remains problematic due to inadequate comprehension of the manner in which starch granules regulate disulfide bond formation within wheat gluten proteins. In order to address this evident gap in the existing literature, this study investigated the effects of different starch granules—including potato, maize, and wheat—on disulfide bond formation of urea-solubilized glutenin (USG). The experimental results indicate that the optimal conditions for enhancing disulfide bonding in potato, maize, and wheat granules (from 0.2162 to 0.5319, 0.3502 and 0.9488 μmol/g, respectively) were as follows: a USG: granule ratio of 3:1 (*w*/*w*), a temperature of 45 °C for 30 min, a USG: granule ratio of 3:1 (*w*/*w*), a temperature of 35 °C for 120 min, a USG: granule ratio of 1:2 (*w*/*w*), a temperature of 25 °C, and a duration of 60 min, respectively. Under low-granule conditions, the possible mechanism was that all granules might leach out predominantly amylopectin (no blue color is observed when attached to an iodine solution) to facilitate disulfide bond formation of USG. Conversely, under high-granule conditions, the interaction between granule proteins may be excessive, potentially leading to the precipitation of amylose (dark blue color is observed when attached to an iodine solution). This process may result in a reduction in disulfide bond contents due to the competitive interaction of water molecules. Spectroscopic and structural analyses further indicated that the attenuation of the nuclear magnetic resonance (NMR) signal of C1 hydroxyl groups of amylopectin/amylose and peptide amide bonds of USG arose from physical entanglement based on the hydrogen bonds between them. Upon interaction between USG and potato/maize starch granules, the X-ray diffraction pattern of USG vanished, and the intramolecular β-sheet conformation was markedly diminished. Collectively, these findings provide a mechanistic foundation for the rational design and optimization of high-fiber, high-quality cereal-based food products.

## 1. Introduction

Chinese wheat-based foods constitute the staple food for people in northern China, including steamed buns, noodles, and doughnuts, etc. [1]. In order to augment the dietary fiber content of baked products, manufacturers frequently incorporate potato or maize flour. However, the incorporation of these coarsely milled cereal flours has the potential to compromise product consistency and, in extreme cases, impair dough structure development and final product integrity. Potato flour has been demonstrated to induce substantial disruption to the gluten network, resulting in a reduction in the content of disulfide bonds and hydrogen bonding density within the dough [2]. Exceeding the recommended incorporation levels of heat-moisture-treated potato starch or microwave-treated potato starch has been shown to result in structural deformation and increased breakage susceptibility in noodles [3]. The incorporation of modified corn flour in excess (i.e., greater than 15% by weight) has been demonstrated to disrupt the integrity of the gluten network structure [4]. In order to mitigate this adverse effect, researchers have investigated various strategies. Bread prepared from composite flour incorporating extruded corn flour exhibits a significantly softer crumb texture, reduced staling rate during storage, greater specific volume, and higher overall sensory evaluation scores compared to bread made with non-extruded corn flour [5]. The incorporation of low-temperature extrusion-modified potato starch, prepared at an extrusion temperature of 60 °C and a moisture content of 42%, has been shown to result in a 22% increase in the specific volume of whole-wheat youtiao [6].

The height of potato–wheat dough during the fermentation process has been shown to improve significantly when hydrocolloids such as hydroxylpropylmethylcellulose, arabic gum, konjac glucomannan and apple pectin are incorporated [7]. The utilization of hydrocolloids in this manner is impractical due to their capacity to modify the sensory characteristics of foodstuffs. The improvement in dough property by extrusion is presumably attributable to amylopectin leaching from starch granules during the treatment process. The urea-soluble glutenin (USG) accounts for 18.26% of the total gluten protein [8]. The glutenin fraction has been demonstrated to play a pivotal role in protein polymerization, a process that is critical to the development of high-quality cereal-based foods [9]. Disulfide bonds have been demonstrated to have a significant impact on the mechanical stability, elasticity, extensibility, and network stability of dough, which is attributed to the formation of macromolecules [10,11]. The interaction of starch–protein leads to higher levels of free thiol groups in whole gluten [12], but the detailed mechanism is difficult to describe because of complexation of gluten. In this paper, purified potato, maize, wheat granules and USG are mixed to investigate the impact of potato, maize, and wheat starch granules on the disulfide bond content of USG, aiming to elucidate the underlying molecular mechanisms governing protein–starch interactions in gluten-based systems. The most significant finding of this study is that the three types of granules modulate disulfide bond cleavage or reformation in USG through the release of amylose/amylopectin.

## 2. Materials and Methods

### 2.1. Materials

Potato (20~22% amylose, 0.04% lipids, 0.05% protein, particle size ~42 μm), maize (26~27% amylose, 0.07% lipids, 0.21% protein, particle size ~14 μm) and wheat (24~25% amylose, 0.8% lipids, 0.25% protein, particle size 38~51 μm) starches were purchased from Henan Enmiao Food Co., Ltd. (Zhengzhou City, China). Gluten powder (9% moisture, 2% lipids, 6% carbohydrates, 83% protein) was from Puyang Qihexiang Co., Ltd. (Puyang City, China). The microbial lipase (20,000 U/g) and alkaline protease (50,000 U/g) utilized in this study were purchased from Beijing Sola Biotechnology Co., Ltd. (Beijing, China). Iodine solution, sodium hydroxide, sodium chloride, and hydrochloric acid were purchased from Tianjin Fengchuan Chemical Reagent Co., Ltd. (Tianjin, China). Ethanol (95%) was procured from Henan Xinheyang Alcohol Co., Ltd. (Jiaozuo City, China). Sodium chloride (analytical reagent containing 99.5% NaCl) was produced by Tianjin Hengxing Chemical Reagent Manufacturing Co., Ltd. (Tianjin, China). Tris-base, glycine (Glycine), disodium EDTA, urea, and DTNB reagents were all from Beijing Solabo Technology Co., Ltd. (Beijing, China).

### 2.2. Purification of Starch Granules

A quantity of 15 g of normal starch was dispersed in 25 mL of deionized water. The mixture was then heated to 80 °C in a water bath, with continuous stirring, until a homogeneous, viscous slurry was formed. Subsequently, the slurry was subjected to a freezing process at a temperature of −20 °C for a duration of 12 h. This was followed by a thawing procedure conducted at ambient temperature. Subsequently, the pH of the resulting suspension was adjusted to 10.0 using 2.0 M of NaOH. Alkaline protease (50,000 U/g; 150 mg) was added to the mixture, and enzymatic hydrolysis was carried out at 55 °C for 6 h. Thereafter, lipase (20,000 U/g; 150 mg) was added to yield a final concentration of 0.1% (*w*/*v*), and hydrolysis was continued at 55 °C for an additional 6 h. The resultant precipitate was collected by centrifugation (3000× *g*, 5 min) to remove soluble amylose or amylopectin. The precipitate was then washed three times with deionized water and once with 65% (*v*/*v*) aqueous ethanol. The purified starch granules were recovered as a wet paste (wet weight: 33–36 g; estimated dry weight: 8–9 g) and subsequently dried to constant mass at 60 °C.

### 2.3. Preparation of USG

A quantity of commercial gluten (400.00 g) was suspended in 65% (*v*/*v*) aqueous ethanol (12 L) and stirred at 35 °C for 3 h. This extraction procedure was repeated twice. The combined suspensions were subjected to a centrifugal process at 4000× *g* for a duration of 5 min at a temperature of 4 °C. This resulted in the formation of a yellow, homogeneous, gel-like precipitate, which was subsequently designated as crude wheat gluten protein (164.80 g). The precipitate was fragmented into small pieces and subsequently solubilized in a saturated urea solution (solid-to-liquid ratio: 1:1, *w*/*v*) under continuous stirring for a period of 6 h at room temperature. Following a centrifugation process (4000× *g*, 5 min, 4 °C), the supernatant—designated as the urea-solubilized wheat gluten protein fraction—was collected. The process of urea crystallization was induced by subjecting the solution to a controlled evaporation of water at a temperature of 20 °C and a humidity level of 50%, corresponding to the range of 15–25 °C and 30–50%, respectively. This process was continued until the observation of complete precipitation of needle- or prism-shaped urea crystals and the completion of desiccation of the protein fraction. The dried residue was washed three times with deionized water to ensure the removal of all residual urea. The resulting purified product that resulted from this process was designated USG. Finally, the USG was dried to constant weight at 60 °C in an air blast drying oven. The protein content of USG was more than 99% (as determined by the Kjeldahl method, GB5009.5-2016 National Food Safety Standard for the Determination of Protein Content in Foods).

### 2.4. Mixture of USG and Granules

USG samples, weighing 15 milligrams each, were dispersed in 12 mL of deionized water to achieve homogeneity. Following this step, the samples were then mixed with three types of purified granules, each derived from potato, maize, and wheat starches. The mixture contained a USG concentration of 1.25 mg/mL. The USG-to-starch mass ratios employed were 3:1, 2:1, 1:1, 1:3, and 1:2. Each mixture was subjected to controlled temperature conditions of 25 °C, 35 °C, and 45 °C for durations of 30, 60, 90, and 120 min, respectively. Subsequent to the incubation period, the samples were subjected to a drying process at a constant temperature of 60 °C in an air blast drying oven, with ambient atmospheric pressure maintained throughout the process. All samples were performed in triplicate (*n* = 3).

### 2.5. Determination of Disulfide Bond Contents

The quantification of free thiol (–SH) and total thiol group contents in the sample was performed spectrophotometrically using 5,5′-dithiobis(2-nitrobenzoic acid) (DTNB), following the method described by [13]. The disulfide bond (S–S) content was subsequently derived from the difference between total and free thiols.

Reagent preparation:

(A) Tris–Glycine buffer (pH 8.0): The tris–glycine buffer (pH 8.0) was prepared by first dissolving 10.418 g of Tris base, 6.756 g of glycine, 2.350 g of guanidine hydrochloride, and 1.489 g of disodium EDTA in distilled water. The volume was then adjusted to 1000 mL.

(B) Tris–Glycine–8 M urea buffer: The following reagents were dissolved in distilled water: 10.418 g of Tris base, 6.756 g of glycine, 1.489 g of disodium EDTA, and 480.48 g of urea. The volume was adjusted to 1000 mL after the addition of 0.05 mL of β-mercaptoethanol.

(C) DTNB working solution (4 mg/mL): Prior to each assay, the substance was prepared by dissolving 4 mg of DTNB that was dissolved in 1 mL of solution A. The solution was then diluted to a 1:1 ratio with the additional solution A to yield a final concentration of 4 mg/mL.

Free thiol quantification: A 15 mg aliquot of the USG sample was dissolved in 5 mL of solution A, yielding a nominal protein concentration of 3 mg/mL. Subsequently, 50 μL of solution C was added to the mixture. The mixture was then incubated at 25 °C for 60 min in the dark. Following this, the mixture was subjected to centrifugation at 13,600× *g* for 10 min. Absorbance of the supernatant was measured at 412 nm against a blank reagent.

The quantification of total thiol is the subject of this text. The experiment was conducted identically, except that solution B replaced solution A to fully reduce disulfide bonds prior to the DTNB reaction.

Quantitative calculations:

The free thiol concentration (in micromoles per gram of protein) was calculated as follows: 73.53 × A_412_ × D/C, where A_412_ was the corrected absorbance at 412 nm (sample minus blank reagent), D was the dilution factor, and C was the protein concentration (g/mL) of the original sample solution.

The concentration of disulfide bonds (μmol/g) was calculated as follows: (Total thiol − Free thiol)/2.

It is important to note that all measurements were performed in triplicate (*n* = 3), and the results are reported as the mean ± standard deviation where applicable.

### 2.6. Fourier-Transform Infrared (FT-IR) Spectroscopy

Secondary structural characteristics were determined by Fourier-transform infrared (FT-IR) spectroscopy, following established protocols [14]. Briefly, dried sample powder was placed on a slide holder, and spectra were acquired in transmission mode using a PerkinElmer Spectrum FT-IR spectrometer (PerkinElmer, Buckinghamshire, UK) at 27 °C. Spectra were recorded over the wavenumber range of 4000–400 cm^−1^ with a resolution of 4 cm^−1^ scans. Characteristic absorption bands corresponding to major secondary structural elements were assigned as follows: α-helix (1646–1661 cm^−1^ and 1295–1330 cm^−1^), β-sheet (1615–1637 cm^−1^ and 1220–1250 cm^−1^), β-turns (1665, 1670, 1675, 1683, and 1688 cm^−1^; 1270–1295 cm^−1^), random coil (1637–1648 cm^−1^), and reverse β-sheet (1670–1695 cm^−1^). Peak deconvolution and quantification were performed using the PeakFit v4.12 software (Systat Software Inc., Chicago, IL, USA), and relative secondary structure contents were calculated from integrated peak areas after baseline correction.

### 2.7. Solid-State ^13^C Nuclear Magnetic Resonance (ssNMR) Spectroscopy

Solid-state ^13^C cross-polarization magic-angle spinning (CP/MAS) NMR spectra were acquired on a JEOL ECZ600R 600 MHz spectrometer (JEOL RESONANCE Inc., Tokyo, Japan). The quantity of sample utilized in this study ranged from 50 to 80 milligrams, which was packed into a 5 mm zirconia rotor. The rotor was then spun at a specific angle, referred to as the “magic angle,” which is defined as 54.7 degrees, using a frequency of 10 kHz. All experiments were conducted at ambient temperature (25 °C) using a ^13^C Larmor frequency of 150.87 MHz, a 90° pulse width of 2.4 µs, a contact time of 2 ms, and a recycle delay of 5 s. Spectra were accumulated over 2048–4096 transients with Hartmann–Hahn cross-polarization.

### 2.8. X-Ray Diffraction (XRD) Analysis

X-ray diffraction patterns were collected using a Rigaku D/MAX-2500 Advance diffractometer (Rigaku Corporation, Tokyo, Japan) equipped with Cu Kα radiation (λ = 1.5418 Å) operated at 40 kV and 200 mA. The data were acquired over a 2θ range of 3–60° with a step size of 0.02° and a dwell time of 0.8 s per step. Samples were meticulously prepared as thin, homogeneous films on zero-background silicon sample holders. They were then maintained at ambient temperature during measurement.

### 2.9. Scanning Electron Microscopy

Scanning electron microscopy (SEM) images were acquired using a JEOL JSM-6490LV scanning electron microscope (JEOL Ltd., Tokyo, Japan). Samples were mounted on carbon tape affixed to an aluminum stub and sputter-coated with a 10 nm layer of carbon. The imaging procedure was executed at an accelerating voltage of 15 kV and a working distance of 10 mm under conditions of high-vacuum. All micrographs were acquired in secondary electron mode, with optimized contrast and brightness settings.

### 2.10. Statistical Analysis

All quantitative data are expressed as the mean ± standard deviation (*n* = 3). The statistical significance among treatment groups was evaluated using one-way analysis of variance (ANOVA), followed by Dunnett’s post hoc test. In this context, *p* < 0.05 is considered statistically significant.

## 3. Results and Discussion

### 3.1. The Effects of Different Granules on Disulfide Bond Contents in USG

Table 1, Table 2 and Table 3 present the effects of various granules on the disulfide bond content of USG.

A comprehensive statistical analysis has revealed that the starch type, the USG-to-granule mass ratio, the mixing temperature, and the mixing duration all exert statistically significant influences on disulfide bond formation. Among the three starch types tested, maize granules exhibited the greatest capacity to disrupt disulfide bonds. This is evidenced by the fact that the highest proportion of samples showing reduced disulfide bond content was found in the maize granule samples (45 out of the total samples). The second-highest proportion of samples showing reduced disulfide bond content was found in the potato granule samples (41), followed by the wheat granule samples (39). The protein-to-granule ratio exerts a substantial influence on disulfide bond content. At ratios of 3:1, 2:1, 1:1, 1:3, and 1:2 (*w*/*w*), the numbers of samples exhibiting reduced disulfide bond contents are as follows: for potato granule—3, 11, 10, 10, and 7; for maize granule—6, 11, 7, 10, and 11; and for wheat granule—9, 11, 7, 10, and 11. The collective analysis of the data indicates that achieving a protein-to-starch granule ratio of 3:1 results in the most consistent enhancement of disulfide bond formation for both potato and maize granules. The mixing temperature also demonstrates a pronounced effect. At temperatures of 25 °C, 35 °C, and 45 °C, the number of samples exhibiting reduced disulfide bond content is as follows: potato granule—11, 15, and 11; maize granule—17, 15, and 13; and wheat granule—9, 17, and 13. The findings of this study suggest that a temperature of 45 °C is optimal for maximizing disulfide bond formation in the presence of potato or maize granules. Furthermore, the duration of mixing has been shown to have a substantial impact on outcomes. Across durations of 30, 60, 90, and 120 min, the numbers of samples exhibiting reduced disulfide bond contents are as follows: potato granule—11, 11, 10, and 7; maize granule—10, 11, 12, and 12; and wheat granule—13, 11, 6, and 9. Thus, 120 min has been identified as the optimal duration for potato granule, while 30 min has been determined to be the optimal duration for maize granule. A comprehensive analysis of all parameters has been conducted to ascertain the optimal conditions for maximizing disulfide bond content. The following parameters have been identified as the most conducive to this end: For the potato granule, the ratio of USG to granules was 3:1. The temperature at which the mixture was prepared was 45 °C. The duration of the mixing process was 30 min (0.5319 μmol/g). The following is the composition of the maize granule: The ratio was determined to be 3:1, with an initial temperature of 35 °C and a duration of 120 min. This resulted in an estimated amount of 0.3714 μmol/g. For the wheat granule, the ratio was determined to be 1:2, with a temperature of 25 °C and a duration of 60 min, resulting in an estimated value of 0.9488 μmol/g.

As illustrated in Figure S1, the visible maximum absorption wavelengths and color development of each starch sample attached to iodine during the interaction process are evident. As shown in Figure S1b,e, the absence of blue coloration in the samples suggests that the primary components extracted from the granules are amylopectin. This phenomenon prompts the formation of disulfide bonds in USG [14]. The dark blue in Figure S1a,d,g indicate that a large amount of water-soluble long-chain double-helix amylose is distributed on the surface of the starch granules. The lighter blue hue in Figure S1c,f,i signifies that within their respective interaction environments, the starch granules release double-helical amylose molecules characterized by shorter chain lengths. Amylose has been observed to disrupt disulfide bonds in USG.

### 3.2. Fourier-Transform Infrared Spectroscopy Analysis

**Figure 1:**
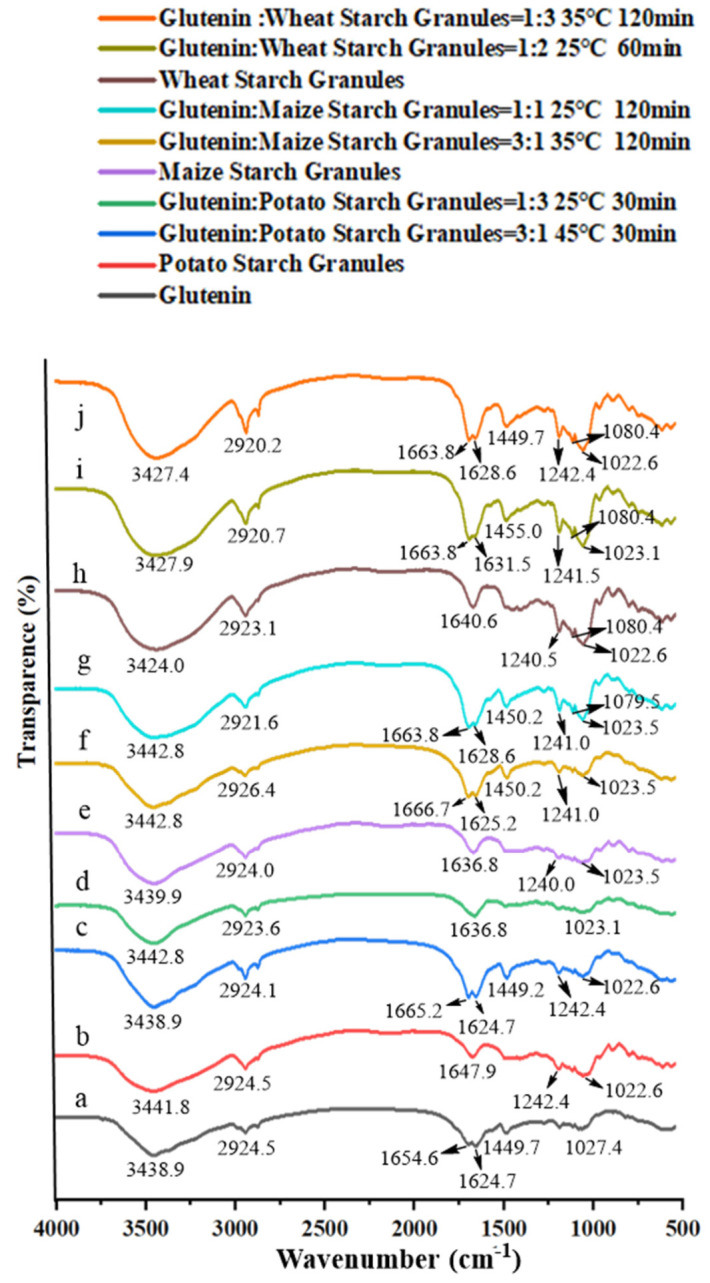
FTIR spectra of USG combined with different granules. (**a**) USG; (**b**) potato starch granule; (**c**) potato starch granule group with higher disulfide bond content; (**d**) potato starch granule group with lower disulfide bond content; (**e**) maize starch granule; (**f**) maize starch granule group with higher disulfide bond content; (**g**) maize starch granule group with lower disulfide bond content; (**h**) wheat starch granule; (**i**) wheat starch granule group with higher disulfide bond content; (**j**) wheat starch granule group with lower disulfide bond content.

Figure 1 presents the Fourier-transform infrared (FTIR) spectra of USG combined with different granules.

Based on established assignments reported in references [15,16], the broad band centered at ~3438 cm^−1^ is attributable to the O–H stretching vibration of starch and/or the N–H stretching vibration of USG. The band at ~2925 cm^−1^ is assigned to the C–H stretching vibration of methylene groups. Two prominent bands at ~1654/1624 cm^−1^ correspond to the C=O stretching vibration of the amide I band. The band at ~1636 cm^−1^ arises from the H–O–H bending vibration of residual water. The band at ~1449 cm^−1^ is assigned to C–N bending vibrations within the amide III band. The band at ~1242 cm^−1^ is attributed to H–N–C bending vibrations (also part of the amide III band). The band at ~1080/1022 cm^−1^ is attributed to C–O stretching vibrations of hydroxyl groups (–COH), which reflect the order or disorder present in the amorphous region of starch. As illustrated in Figure 1a, two distinct spectroscopic features differentiate USG from other gluten protein samples [15]. First, the amide I band exhibits a shoulder peak; second, the amide II band shows markedly attenuated absorbance. These spectral alterations are likely attributable to two interrelated factors: (i) the thermal hydration protocol employed during protein drying at 60 °C, and (ii) partial disruption of intermolecular hydrogen bonds within USG aggregates. This phenomenon is corroborated by prior studies [15]. A comparison of Figure 1b,e,h reveals residual protein deposition on the surfaces of all three starch-based granules, as evidenced by a characteristic absorption band near 1242 cm^−1^. Notably, the medium band at 1636.8 cm^−1^ in Figure 1e for water molecules indicates that the reduced water-holding capacity of maize starch granules may be attributed to the presence of surface-bound proteins, in contrast to other starch types. A more thorough examination of Figure 1b–d might demonstrate a discernible correlation between the hydration behavior of proteins and the concentration of potato starch granules. At a low granule concentration (see Figure 1c), USG might exhibit enhanced water absorption, as evidenced by increased peak intensities at ~1654/1624 cm^−1^, which likely promotes the formation of the intramolecular disulfide bonds in Table 4. In contrast, at an elevated granule concentration (see Figure 1d), the amide I band vanishes entirely, resulting in the sole presence of the characteristic water H–O–H bending vibration signal. The predominant mechanism underlying disulfide bond depletion in USG might be characterized by the pronounced hygroscopicity of surface-adsorbed protein on potato starch granules. Furthermore, the protein exhibits a propensity to localize on smaller starch granules. As illustrated in Figure 1e–g, maize starch granules appear to demonstrate effects of a qualitatively similar nature at low concentrations (Figure 1f). The enhanced disulfide bond formation observed in this study parallels that observed with potato starch. However, at elevated concentrations (see Figure 1g), significant increases become evident in two distinct regions. The first is the absorption band near 1080 cm^−1^, and the second is the H–N–C bending vibration of the amide III band. These changes are plausibly linked to mechanical rupture and subsequent release of amylose from swollen maize starch granules during the mixing process [17]. Amylose’s strong water-binding properties enable its release, which functions in a competitive manner to sequester water from the USG phase. This process has the potential to induce a structural reorganization of the USG, shifting from an intramolecular β-sheet configuration to an intermolecular β-sheet and random coil conformation. Concurrently, this process may result in the cleavage of disulfide bonds. As demonstrated in Figure 1h–j, a pre-existing absorption feature emerges at 1080 cm^−1^ in the wheat starch granule–USG mixture. This finding indicates that the application of enzymes, such as proteases and lipases, during the pretreatment process may result in the partial degradation of the starch matrix, thereby releasing amylopectin prior to the blending stage. As the concentration of wheat starch granules is low (see Figure 1i), it is likely that the elevated amylopectin release probably promotes disulfide bond formation. This finding is consistent with recent research indicating that wheat-derived amylopectin can act as a structural modulator, enhancing crosslinking in alkali-soluble glutenin [14]. Conversely, at a high microsphere concentration (Figure 1j), excessive amylose release might dominate the interaction, leading to progressive destabilization of disulfide bonds and a concomitant slight reduction in the amide III H–N–C bending vibration intensity. Because several bands, such as amide II, overlap considerably, accurate information from the IR spectra is limited.

As illustrated in Table 4, the secondary structural composition of USG was examined before and after interaction with three distinct starch granule types, including wheat, potato, and maize starch. Prior to interaction, the secondary structure distribution is as follows: α-helix (26%), intermolecular β-sheet (2%), intramolecular β-sheet (39%), β-turn (33%), and random coil (0%). This profile deviates from previously reported values for native or conventionally isolated gluten fractions [16], potentially attributable to differences in sample preparation—particularly drying methodologies. Notably, the random coil conformation is exclusively observed in freeze-dried samples, possibly suggesting its sensitivity to dehydration conditions. Despite the administration of protease treatment, trace amounts of residual protein are detected on the surfaces of all three starch granules. However, their secondary structural profiles differed markedly. Specifically, proteins adsorbed onto wheat starch exhibit relatively high α-helix content; those bound to potato starch are predominantly enriched in intermolecular β-sheet structures; and proteins associated with maize starch display elevated levels of both intermolecular and intramolecular β-sheet conformations. Following interaction, wheat starch granules have been observed to induce a substantial increase in disulfide bond formation within USG. In contrast, potato and maize starch granules suppress disulfide bond formation and induce a pronounced reduction in intramolecular β-sheet content—from 39% in the unbound control to 11% and 13%, respectively. These findings suggest that potato and maize starch granules might facilitate partial unfolding of intramolecular β-sheet in USG, thereby disrupting pre-existing disulfide crosslinks.

### 3.3. ^13^C Solid-State NMR Analysis

**Figure 2:**
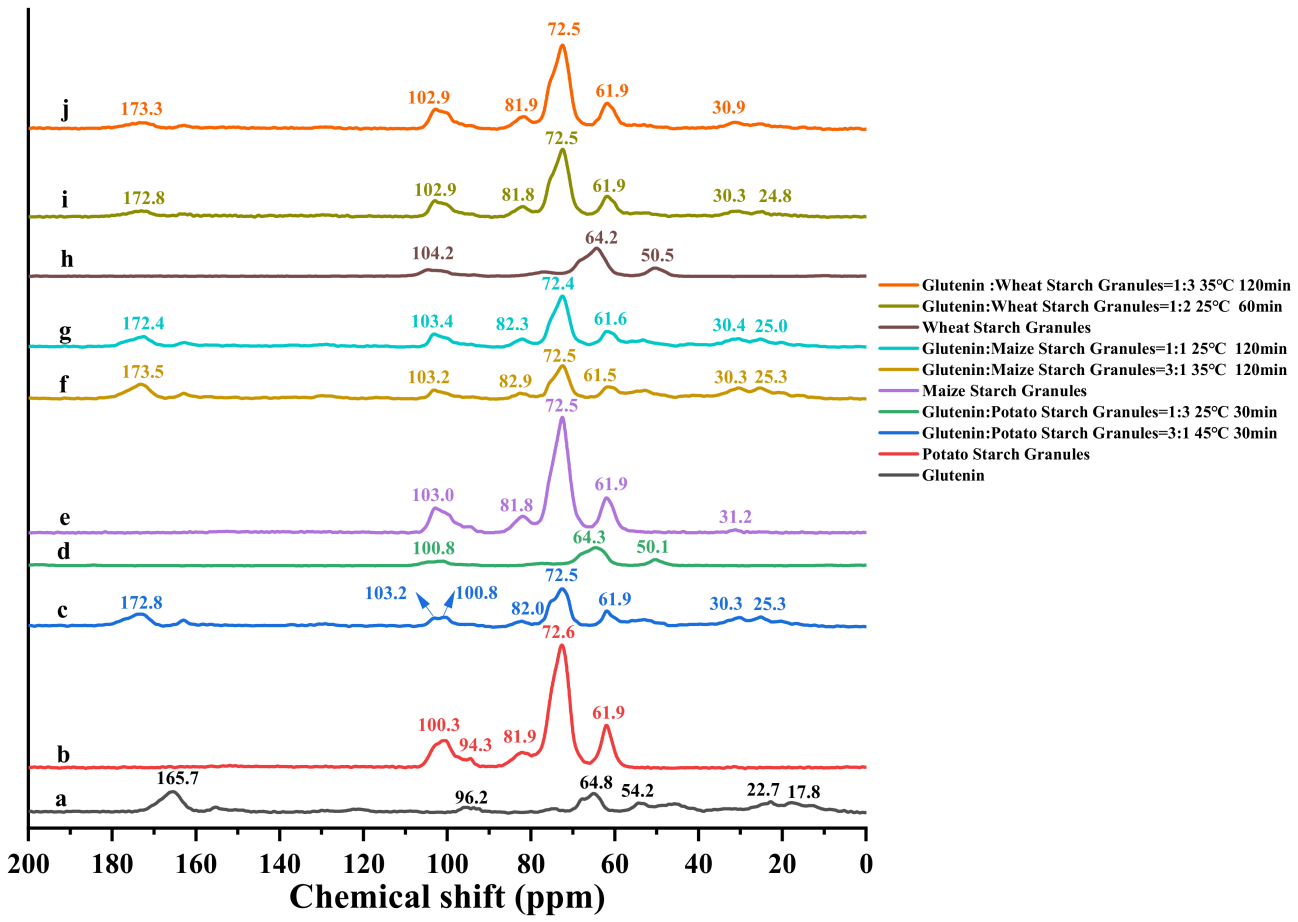
^13^C solid-state NMR spectra of USG combined with different granules. (**a**) USG; (**b**) potato starch granule; (**c**) potato starch granule group with higher disulfide bond content; (**d**) potato starch granule group with lower disulfide bond content; (**e**) maize starch granule; (**f**) maize starch granule group with higher disulfide bond content; (**g**) maize starch granule group with lower disulfide bond content; (**h**) wheat starch granule; (**i**) wheat starch granule group with higher disulfide bond content; (**j**) wheat starch granule group with lower disulfide bond content.

According to the references [16,18,19,20,21], the resonances of USG before and after being bound to different granules in Figure 2 are assigned as follows: ~173 ppm (backbone C=O of amido bond), 165.7 ppm (Y_ζ_ of Tyr), ~103/100 ppm (C1 of starch), ~82 ppm (C4 of starch), ~72 ppm (C2, 3, 5 of oligosaccharides in the amorphous region), ~62/64 ppm (C6 of oligosaccharides or starch), ~54/50 ppm (Q_α_ of Gln), 30 ppm (Q_γ_ of Gln, Pβ of Pro), ~25/23 ppm (P_γ_ of Pro) and 17.8 ppm (methyl L_δ_ of Leu, T_γ_ of Thr). The observation of the resonance at 96.2/64.8 ppm in USG in Figure 2a suggests a potential combination of USG with polysaccharides. The absence of characteristic amide bond signals and polysaccharide C1/C2/C3/C5 resonances in Figure 2a suggests the potential for physical entanglement based on hydrogen bonds between them. Notably, the C6 resonance of polysaccharide units and the P_γ_ resonance of proline in Figure 2a undergo opposite chemical shift changes (downfield and upfield, respectively). These changes are consistent with the presence of intermolecular hydrogen bonds between these sites. As shown in Figure 2b,e,h, the application of enzymes, specifically protease and lipase, results in the effective removal of surface-associated protein from potato starch granules. In contrast, residual protein is observed in maize granules (31.2 ppm, a weak signal in Figure 2e) and is particularly abundant in wheat starch granules (50.5 ppm, a weak signal in Figure 2h). A comparison of Figure 2b,c indicates that under conditions of low potato starch granule concentration and elevated mixing temperature, disulfide bond formation in USG is enhanced. Concurrently, distinct NMR signals attributable to protein amide bonds and amylopectin C1 (103.2 ppm) appear. These signals indicate partial granule rupture and release of potato amylopectin. The released amylopectin interacts with USG, inducing a conformational transition from β-turn to α-helix and intramolecular β-sheet structures (Table 4), thereby facilitating disulfide crosslinking. In contrast, as illustrated in Figure 2b,d, the combination of elevated potato starch granule concentration coupled with low-temperature processing yields only a weak USG-derived signal at 50.1 ppm. Notably, resonances attributed to starch C2–C5 become undetectable. The manifestation of a diminished peak at 100.8 ppm in Figure 2d might suggest extensive granule disruption and amylose releases. It has been demonstrated that upon interaction with USG, these amyloses might promote a shift in the intramolecular β-sheet of USG to intermolecular β-sheet and α-helix conformations. Concurrently, there is a decrease in the intramolecular disulfide bonds in USG. The higher water absorption capacity of potato amylose leads to an increase in the density of USG and the disappearance of nuclear magnetic vibration. As illustrated in Figure 2e,f, when the concentration of maize starch granules is low, there is an enhancement of the amide bond signal upon interaction with USG. Concurrently, the amylopectin C1 resonance diminishes, suggesting an interaction between these two sites. At elevated temperatures, this interaction drives the same secondary structural transition (β-turn → α-helix + intramolecular β-sheet; Table 4) and enhances disulfide bond formation of USG. Conversely, Figure 2e,g show that high maize starch granules attenuate the amide bond signal while amplifying starch-derived resonances. The underlying mechanism is analogous to that of potato starch granules. The low-temperature processing of abundant granules has been shown to favor the release of amylose. This, in turn, has been demonstrated to competitively interfere with the intramolecular disulfide stabilization in USG (see Table 4). Indeed, low-temperature conditions have been shown to facilitate the leaching of amylose from both potato and maize starch granules. Finally, Figure 2h–j indicate that the NMR profiles of wheat starch granule–USG interactions closely resemble those of maize starch, with the exception of a marginally higher intensity at the C4 resonance. This finding potentially reflects the higher side chains in wheat amylose. Crucially, in contrast to the interaction of potato or maize starch granules at high temperatures, the low-temperature interaction with wheat starch granules leads to the greater amylopectin release in Figure 2i. This phenomenon promotes the formation of the intermolecular disulfide bonds in USG in Table 4. An excess of amylose release, as depicted in Figure 2j, results in a subsequent reduction in disulfide bond contents in USG.

### 3.4. X-Ray Diffraction Analysis

**Figure 3:**
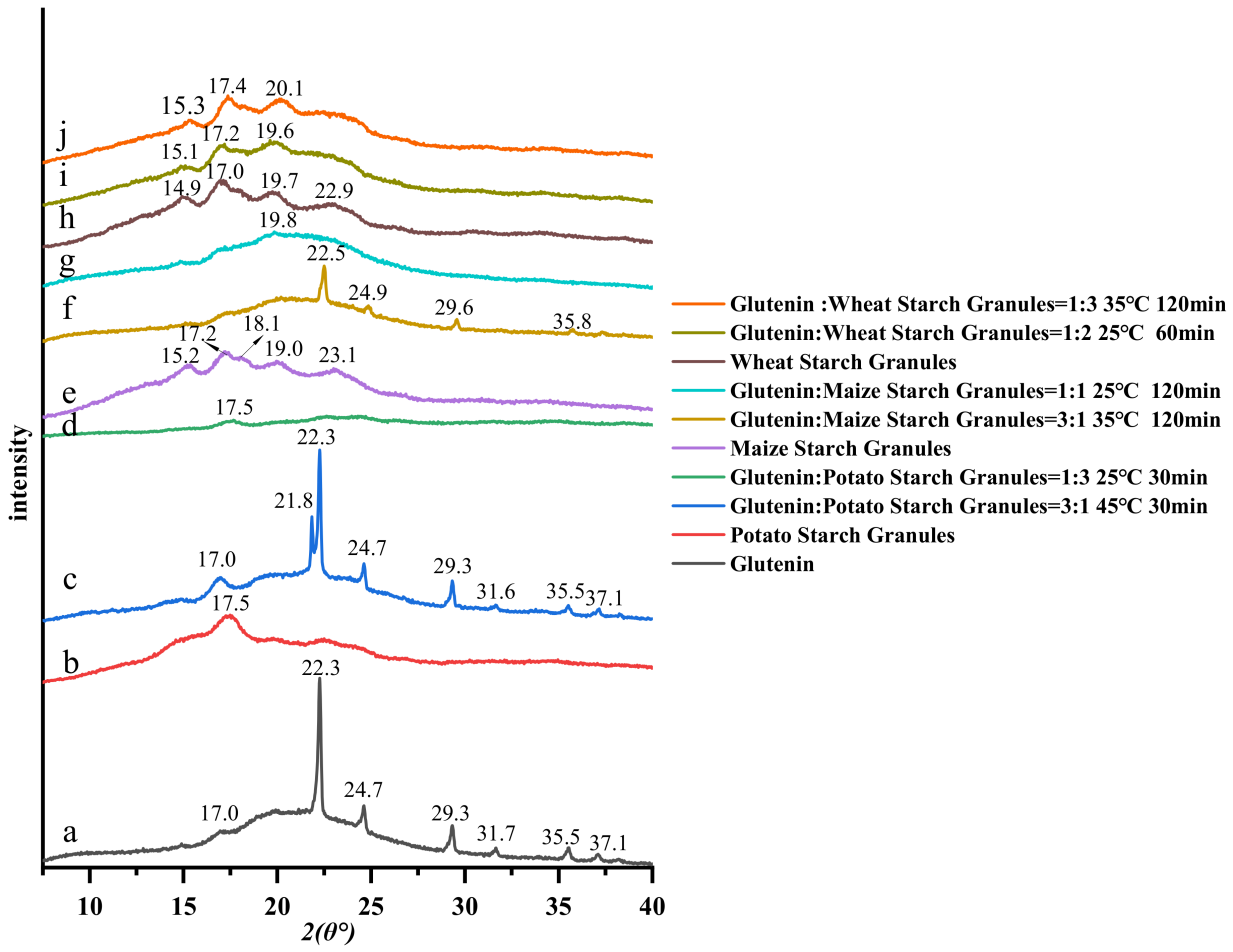
X-ray diffraction of USG and its complex with different granules. (**a**) USG; (**b**) potato starch granule; (**c**) potato starch granule group with higher disulfide bond content; (**d**) potato starch granule group with lower disulfide bond content; (**e**) maize starch granule; (**f**) maize starch granule group with higher disulfide bond content; (**g**) maize starch granule group with lower disulfide bond content; (**h**) wheat starch granule; (**i**) wheat starch granule group with higher disulfide bond content; (**j**) wheat starch granule group with lower disulfide bond content.

As illustrated in Figure 3, the X-ray diffraction (XRD) patterns of USG and the three types of enzymatically hydrolyzed starch granules (potato, maize, and wheat) were examined before and after their interaction. The XRD pattern of USG exhibits characteristic diffraction peaks at 2θ = 17.0° (weak), 22.3° (strong), 24.7° (medium), 29.3° (medium), 31.7° (weak), 35.5° (medium), and 37.1° (weak), which align well with previously reported structural features of gluten proteins [16]. In contrast, the enzymatically treated starch granules display distinct peak profiles: potato granules show a single medium-intensity peak at 17.5°; maize starch granules exhibit peaks at 15.2° (medium), 17.2° (medium), 18.1° (weak), 19.0° (weak), and 23.1° (weak); and wheat starch granules display peaks at 14.9° (weak), 17.0° (medium), 19.7° (medium), and 22.9° (weak). These patterns deviate from those typically observed for native or plasticized starch films reported in the literature [22], suggesting that the structure has been modified by protease and lipase hydrolysis. The attenuation or disappearance of certain starch-derived diffraction peaks is attributable to surface-associated protein–lipid complexes prior to enzymatic treatment. Specifically, in potato starch granules, proteins and lipids are predominantly localized on the surface, resulting in near-complete loss of crystalline XRD signatures after enzymatic hydrolysis, leaving only one residual peak. In contrast, maize and wheat starch granules exhibit a more uniform distribution of proteins and lipids across both surface and interior regions, leading to partial retention of diffraction peaks. It is noteworthy that the broad peak centered at approximately 18° observed in the maize and wheat starch samples may originate from surface-bound protein–lipid assemblies rather than intrinsic starch crystallinity. Interaction with USG results in a subsequent evolution of the XRD behavior. The addition of potato starch granules with a low mass ratio (see Figure 3c) results in the emergence of a new shoulder peak that emerges at 21.8° (corresponding to the interplanar spacing of 4.2 Å), as reported in the literature [23]. This observation indicates the formation of glutenin nanocrystals. As the potato starch loading increases (see Figure 3d), the overall XRD pattern is substantially suppressed, indicating a disruption of the crystalline order in both large potato starch granules and USG. However, small potato granules maintain their structural integrity, albeit without detectable crystallinity. A similar trend is observed for maize starch granules, though the crystalline structure of large maize starch granules is also fully disrupted upon interaction, as evidenced by the absence of starch-specific peaks in Figure 3f. Interaction with wheat starch granules (Figure 3h–j) results in a significant reduction in the 22.9° peak, indicating a direct association between USG and the starch chains responsible for this reflection. This finding is consistent with molecular-level complexation rather than mere physical mixing.

### 3.5. SEM

**Figure 4:**
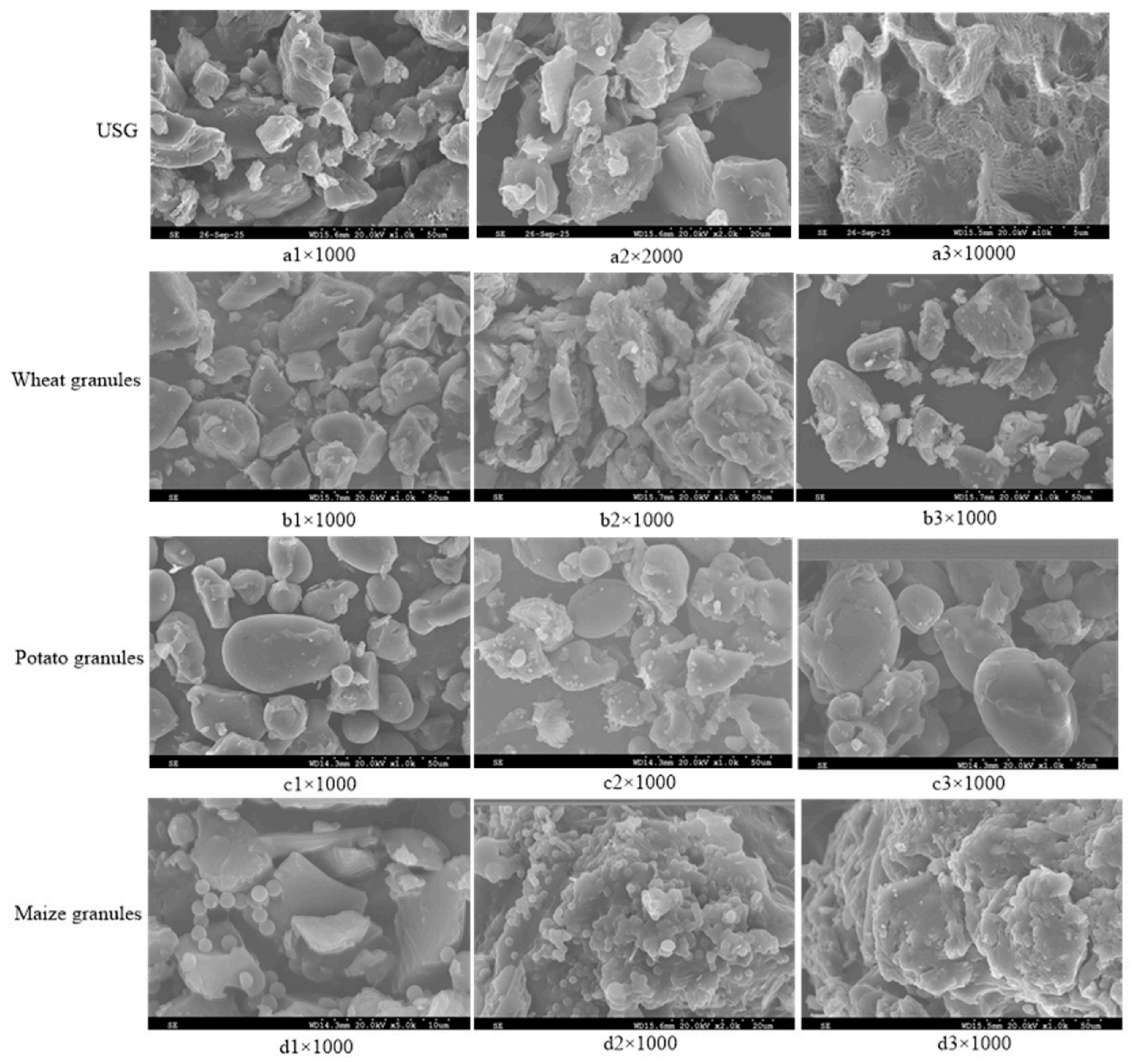
SEM spectra of USG and its complex with different granules: (**a1**–**a3**) USG at different magnifications; (**b1**) wheat granules; (**b2**) wheat granules + USG complex with highest disulfide bonds (0.9488 μmol/g); (**b3**): wheat granules + USG complex with less disulfide bonds (0.123 μmol/g); (**c1**) potato granules; (**c2**) potato granules + USG complex with highest disulfide bonds (0.5319 μmol/g); (**c3**) potato granules + USG complex with less disulfide bonds (0.0176 μmol/g); (**d1**) maize granules; (**d2**) maize granules + USG complex with highest disulfide bonds (0.3714 μmol/g); (**d3**) maize granules + USG complex with less disulfide bonds (0.0383 μmol/g).

As illustrated in Figure 4, scanning electron microscopy (SEM) images of USG and the three types of starch granules (wheat, potato, and maize) were obtained before and after mixing. As shown in Figure 4(a1–a3), USG forms ribbon-like aggregates that are distinguished by their smooth surfaces and a dense, porous network structure with uniformly distributed microvoids. In contrast, native wheat starch granules exhibit irregular, polyhedral morphology (Figure 4(b1)), with diameters ranging from ~50 to 300 μm, consistent with previously reported values [14]. At low wheat starch granule loading (Figure 4(b2)), enhanced disulfide bond formation in USG is observed, resulting in a compact, homogeneous composite structure wherein the interfacial distinction between protein and starch phases becomes indistinct. This finding indicates that the application of mechanical and/or interfacial stresses during the mixing process may induce partial disruption of the starch granule architecture. In the initial phase of protease and lipase purification, the surface proteins and lipids of the starch granules should be eliminated. The surface structure of the wheat starch granules may contain a reduced amount of cellulose and pectin substances, with proteins and lipids forming the primary components of the coating structure. The interaction process between the two elements might result in the rupture of the starch granules, leading to the release of wheat amylopectin. This, in turn, might promote the formation of disulfide bonds in urea-soluble wheat gluten proteins [14]. Conversely, at high wheat starch granule loading (Figure 4(b3)), a greater number of granule disruption might occur, amylose release might be enhanced, and it might reduce the disulfide bond density of USG. The granules of potato starch exhibit an ellipsoidal morphology, with diameters ranging from approximately 50 to 500 micrometers (Figure 4(c1)). As the temperature increases, the loading remains low (see Figure 4(c2)). Numerous small granules interpenetrate the USG matrix. The amylopectin leached from the larger granules at higher temperatures (45 °C) increases the disulfide bond content in USG. The addition of substantial quantities of potato starch granules (Figure 4(c3)) has been observed to result in a reduction in the content of disulfide bonds in USG. This phenomenon occurs when amylose precipitated from smaller starch granules is subjected to a lower temperature (25 °C). The susceptibility of potato starch granules to structural perturbation may be attributed to their elevated surface-associated fiber and pectin content, a feature that has been corroborated by the prior literature [24].

A hitherto unreported bimodal morphology is exhibited by maize starch granules, which consist of spherical small granules and amorphous, irregularly shaped large granules (see Figure 4(d1)). This heterogeneity is likely attributable to incomplete dissociation during the enzymatic pretreatment process, as evidenced by the absence of similar morphology in other studies [25,26,27]. The interaction behavior exhibited by these samples parallels that of potato starch. Specifically, at 35 °C, a low granule loading enhances USG disulfide bonding (Figure 4(d2)). Conversely, at 25 °C, a high loading suppresses this bonding (Figure 4(d3)). However, it is crucial to note that both small and large maize starch granules might undergo structural disruption and release maize amylopectin during the mixing process. Although less effective than wheat amylopectin, maize amylopectin might still promote disulfide bond formation in USG.

## 4. Conclusions

The findings in the paper suggest an association between starch granule characteristics and changes in USG structure under the experimental conditions studied. Specifically, under relatively high temperatures (35/45 °C) and low starch-addition ratios (USG:granule = 3:1), both potato and maize starch granules promote disulfide bond formation in USG. In contrast, at reduced temperatures (25 °C) and elevated starch-addition ratios (USG:granule = 1:3 or 1:1), these granules exhibit a capacity to impede disulfide bond formation. In a similar manner, the regulatory effect of wheat starch granules is temperature- and ratio-dependent. Disulfide bond formation in USG is enhanced at 25 °C with a low addition ratio (USG:granule = 1:2). However, it is inhibited at 35 °C with a high addition ratio (USG:granule = 1:3). The findings, when considered collectively, demonstrate that starch granule type, incubation temperature, and USG-to-granule mass ratio might jointly govern amylopectin/amylose release. This, in turn, fine-tunes the redox-mediated structural reorganization of USG. The USG–granule system is a simplified model to investigate the effects of granules on disulfide bond formation of gluten. In practical applications, gluten strength can be enhanced by incorporating a small quantity of potato or corn flour and extending the dough kneading duration.

## Figures and Tables

**Table 1:** Effects of potato granule addition on disulfide bond contents in USG (μmol/g).

Ratio of USG and Starch Granule	Mixing Temperature (°C)	Mixing Time (min)			
		30	60	90	120
1:1	25	0.0592 ± 0.0038 **	0.1262 ± 0.0124 *	0.1268 ± 0.0038 *	0.5042 ± 0.0447 *
	35	0.1174 ± 0.0088	0.1547 ± 0.0040 **	0.1035 ± 0.0114 **	0.1118 ± 0.0089 **
	45	0.0954 ± 0.0039 **	0.1635 ± 0.0098 *	0.2816 ± 0.003 **	0.0903 ± 0.0105 **
1:2	25	0.1031 ± 0.0062 **	0.1934 ± 0.0683	0.0432 ± 0.0116 **	0.0875 ± 0.0752
	35	0.2810 ± 0.0172 *	0.0540 ± 0.0106 **	0.1765 ± 0.0103	0.0626 ± 0.0078 **
	45	0.1001 ± 0.0203 *	0.1531 ± 0.0217 *	0.0834 ± 0.0117 **	0.2509 ± 0.0114 **
1:3	25	0.0176 ± 0.0098 **	0.1072 ± 0.0101 **	0.2471 ± 0.0051 *	0.2358 ± 0.0142 *
	35	0.4143 ± 0.0741 *	0.1041 ± 0.0095 **	0.3302 ± 0.0000 **	0.3569 ± 0.0087 **
	45	0.1388 ± 0.0253	0.1141 ± 0.0260 *	0.0549 ± 0.0084 **	0.1141 ± 0.0075 **
2:1	25	0.1400 ± 0.0967 **	0.1354 ± 0.0043 **	0.1411 ± 0.1773	0.3443 ± 0.0251 *
	35	0.1362 ± 0.0061 **	0.1358 ± 0.0022 **	0.1868 ± 0.0162	0.1057 ± 0.0005 **
	45	0.0981 ± 0.0028 **	0.1586 ± 0.000 *	0.2116 ± 0.0008	0.1678 ± 0.0064 *
3:1	25	0.4137 ± 0.0042 **	0.3750 ± 0. 0407 *	0.3292 ± 0.0150 *	0.2593 ± 0.0027 *
	35	0.0892 ± 0.0082 **	0.1121 ± 0.0015 **	0.1650 ± 0.0140 *	0.3276 ± 0.0321 *
	45	0.5319 ± 0.0243 **	0.4931 ± 0.0013 **	0.2806 ± 0.0026 **	0.5021 ± 0.0130 **

Note: USG: 0.2162 ± 0.0086, * *p* ˂ 0.05, ** *p* ˂ 0.01.

**Table 2:** Effects of maize granule addition on disulfide bond contents in USG (μmol/g).

Ratio of USG and Starch Granule	Mixing Temperature (°C)	Mixing Time (min)			
		30	60	90	120
1:1	25	0.2004 ± 0.0299	0.0967 ± 0.0316 *	0.1053 ± 0.0074 **	0.0383 ± 0.0113 **
	35	0.2427 ± 0.0011 *	0.3440 ± 0.0942	0.0544 ± 0.0076 **	0.2033 ± 0.0074 **
	45	0.3502 ± 0.0040 **	0.2688 ± 0.0017 *	0.2162 ± 0.0031	0.0906 ± 0.0440
1:2	25	0.0819 ± 0.0071 **	0.1249 ± 0.0245 *	0.2234 ± 0.0054	0.2450 ± 0.0014 *
	35	0.2082 ± 0.0344	0.1026 ± 0.0088 **	0.1864 ± 0.0067	0.1205 ± 0.0159 *
	45	0.1200 ± 0.0039 **	0.1533 ± 0.0084 *	0.1443 ± 0.0172 *	0.1831 ± 0.0010 *
1:3	25	0.0740 ± 0.0027 **	0.1539 ± 0.0080 *	0.2937 ± 0.0159 *	0.1136 ± 0.0137 *
	35	0.2060 ± 0.0033	0.2134 ± 0.0214	0.1377 ± 0.0118 *	0.1089 ± 0.0143 *
	45	0.2066 ± 0.0023 *	0.2233 ± 0.0073 **	0.1330 ± 0.000 **	0.1755 ± 0.0097 *
2:1	25	0.1077 ± 0.0172 *	0.0414 ± 0.0091 **	0.0797 ± 0.0084 **	0.0851 ± 0.0181 *
	35	0.2039 ± 0.0034	0.1224 ± 0.0182 *	0.2104 ± 0.0053	0.0901 ± 0.0069 **
	45	0.2582 ± 0.0231	0.1969 ± 0.0115	0.0910 ± 0.0065 **	0.0905 ± 0.0066 **
3:1	25	0.0831 ± 0.0126 **	0.1018 ± 0. 0287 *	0.0707 ± 0.0152 **	0.1148 ± 0.0064 **
	35	0.3447 ± 0.0118 **	0.2913 ± 0.0047 **	0.1672 ± 0.0007 *	0.3714 ± 0.0006 **
	45	0.2751 ± 0.0089 *	0.1530 ± 0.0089 *	0.3068 ± 0.0001 **	0.2427 ± 0.0012 *

Note: USG: 0.2162 ± 0.0086, * *p* ˂ 0.05, ** *p* ˂ 0.01.

**Table 3:** Effects of wheat granule addition on disulfide bond contents in USG (μmol/g).

Ratio of USG and Starch Granule	Mixing Temperature (°C)	Mixing Time (min)			
		30	60	90	120
1:1	25	0.3652 ± 0.0237 *	0.3155 ± 0.0017 **	0.4361 ± 0.0071 **	0.1389 ± 0.0059 **
	35	0.0713 ± 0.0007 **	0.1298 ± 0.0019 **	0.3168 ± 0.0062 **	0.2578 ± 0.0007 **
	45	0.1089 ± 0.0021 **	0.1057 ± 0.0012 **	0.1200 ± 0.0161 *	0.2313 ± 0.0009
1:2	25	0.4017 ± 0.0039 **	0.9488 ± 0.0037 **	0.7413 ± 0.0129 **	0.4241 ± 0.0040 **
	35	0.0254 ± 0.0017 **	0.1821 ± 0.0043 *	0.1857 ± 0.0095	0.0447 ± 0.0024 **
	45	0.0315 ± 0.0103 **	0.0991 ± 0.0014 **	0.4199 ± 0.0042 **	0.2270 ± 0.0090
1:3	25	0.0987 ± 0.0045 **	0.1911 ± 0.0038	0.3361 ± 0.0087 *	0.1565 ± 0.0133 *
	35	0.0493 ± 0.0022 **	0.0949 ± 0.0045 **	0.1138 ± 0.0223 *	0.0091 ± 0.0077 **
	45	0.1196 ± 0.0084 **	0.1145 ± 0.0114 **	0.4137 ± 0.0042 **	0.4657 ± 0.0174 **
2:1	25	0.0880 ± 0.0250 *	0.2319 ± 0.0160	0.2919 ± 0.0172 *	0.1137 ± 0.0079 **
	35	0.1684 ± 0.0040 *	0.1418 ± 0.0017 **	0.1116 ± 0.0258 *	0.1568 ± 0.0065 **
	45	0.1232 ± 0.0066 **	0.1083 ± 0.0042 **	0.1759 ± 0.0061 *	03936 ± 0.0094 **
3:1	25	0.0989 ± 0.0001 **	0.1078 ± 0.0008 **	0.2857 ± 0.0075 *	0.2127 ± 0.0055
	35	0.0436 ± 0.0059 **	0.1096 ± 0.0010 **	0.2311 ± 0.0012	0.0123 ± 0.0008 **
	45	0.1336 ± 0.0150 *	0.2326 ± 0.0003	0.1218 ± 0.0184 *	0.0837 ± 0.0163 **

Note: USG: 0.2162 ± 0.0086, * *p* ˂ 0.05, ** *p* ˂ 0.01.

**Table 4:** The secondary structure of USG combined with and without wheat, potato and maize granules (%).

Sample	α-Helix	Intermolecular β-Sheet	Intramolecular Aggregation Extended β-Sheet	β-Turn	Random Coils
Glutenin	26	2	39	33	0
Potato Starch Granules	0	91	9	0	0
Glutenin:Potato Starch Granules = 3:1 45 °C 30 min	33	5	62	0	0
Glutenin:Potato Starch Granules = 1:3 25 °C 30 min	34	27	11	29	0
Maize Starch Granules	11	34	31	16	8
Glutenin:Maize Starch Granules = 3:1 35 °C 120 min	35	3	62	0	0
Glutenin:Maize Starch Granules = 1:1 25 °C 120 min	29	29	13	0	29
Wheat Starch Granules	46	22	0	0	32
Glutenin:Wheat Starch Granules = 1:2 25 °C 60 min	17	45	0	0	38
Glutenin:Wheat Starch Granules = 1:3 35 °C 120 min	18	30	14	38	0

## Data Availability

The original contributions presented in this study are included in the article. Further inquiries can be directed to the corresponding author.

## Supplementary Materials

The following supporting information can be downloaded at: https://www.mdpi.com/article/10.3390/foods15152732/s1, Figure S1: The visible maximum absorption wavelengths and color development of each starch sample attached to iodine in the interaction process. Note: (a) potato starch granule, (b) potato starch granule group with higher disulfide bond content, (c) potato starch granule group with lower disulfide bond content, (d) maize starch granule, (e) maize starch granule group with higher disulfide bond content, (f) maize starch granule group with lower disulfide bond content, (g) wheat starch granule, (h) wheat starch granule group with higher disulfide bond content, (i) wheat starch granule group with lower disulfide bond content.
