# Supplementary material for: The Impact of Purified Granules Sourced from Potato, Maize and Wheat on Disulfide Bond Formation in Urea-Solubilized Glutenin
Source: Foods. 2026 Aug 4;15(15):2732. doi: 10.3390/foods15152732 (PMC13465296; doi:10.3390/foods15152732)
Supplement: Supplementary file 1 [file foods-15-02732-s001.zip › foods-4447928-supplementary.pdf]

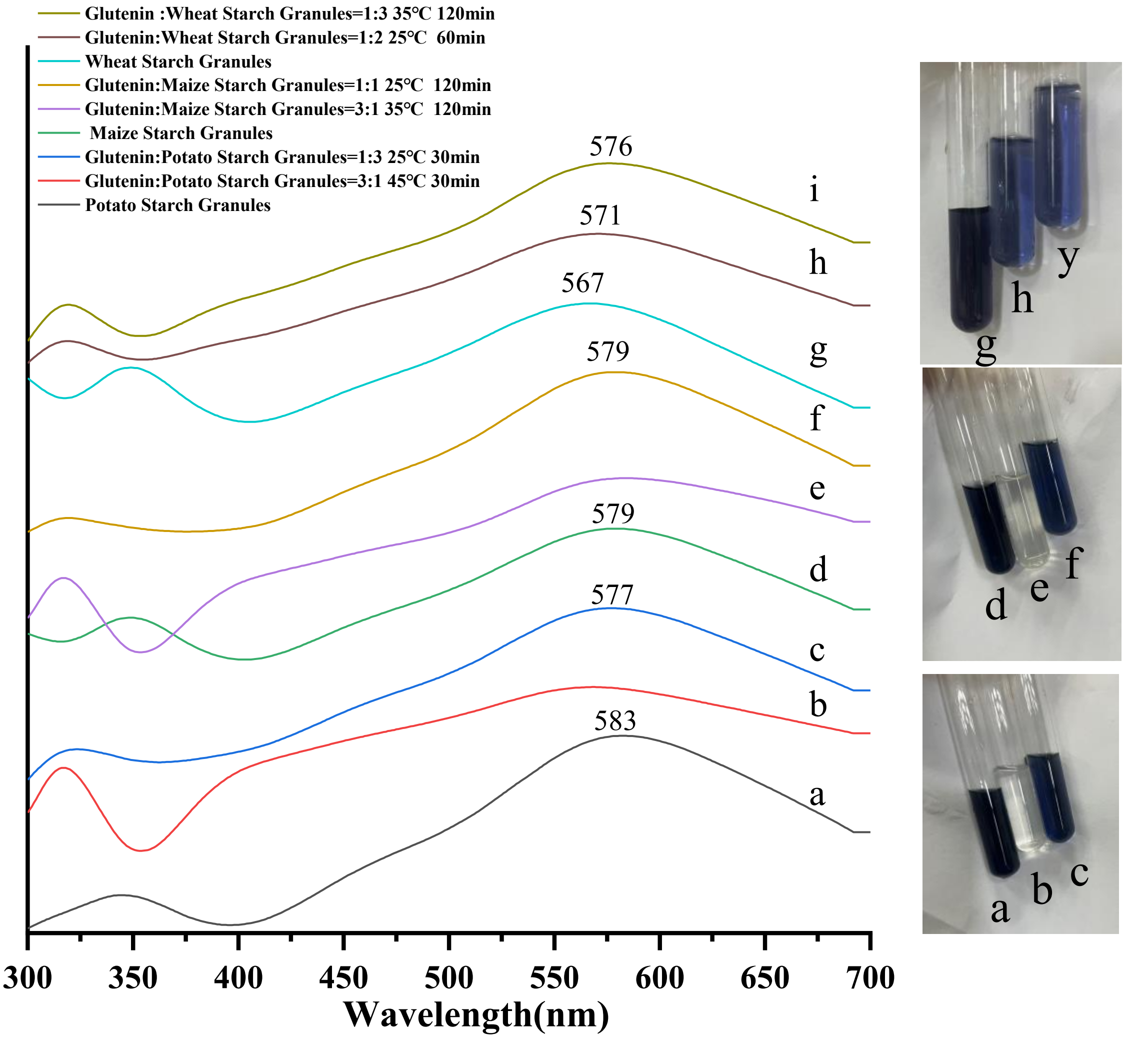

Figure. S1 The visible maximum absorption wavelengths and color development of each starch sample attached to iodine in the interaction process. (**a**)potato starch granule, (**b**) potato starch granule group with higher disulfide bond content,(**c**) potato starch granule group with lower disulfide bond content, (**d**)maize starch granule, (**e**) maize starch granule group with higher disulfide bond content, (**f**)maize starch granule group with lower disulfide bond content,(**g**)wheat starch granule, (**h**)wheat starch granule group with higher disulfide bond content, (**i**)wheat starch granule group with lower disulfide bond content.
